# Treatment patterns and prognosis of patients with clear cell adenocarcinoma of the cervix: a population-based cohort study

**DOI:** 10.1097/JS9.0000000000001997

**Published:** 2024-08-02

**Authors:** Jing Li, Huimin Qiao, Yang Yang, Lan Wu, Dongdong Xu, Zhongqiu Lin, Huaiwu Lu

**Affiliations:** a The Department of Gynecological Oncology, Sun Yat-sen Memorial Hospital of Sun Yat-Sen University; b Guangdong Provincial Key Laboratory of Malignant Tumor Epigenetics and Gene Regulation, Sun Yat-sen Memorial Hospital, Sun Yat-sen University; c The Department of Gynecology, Sun Yat-sen Memorial Hospital of Sun Yat-Sen University, Guangzhou; d Bao’an Center Hospital of Shenzhen, Shenzhen; e Affiliated Jiangmen Traditional Chinese Medicine Hospital of Jinan University, Jinan University, Jiangmen; f National Cancer Center/National Clinical Research Center for Cancer/Cancer Hospital and Shenzhen Hospital, Chinese Academy of Medical Sciences and Peking Union Medical College, Shenzhen, People’s Republic of China

**Keywords:** clear cell carcinoma of the cervix, overall survival, surgery, propensity score matching, SEER

## Abstract

**Objectives:**

To describe treatment patterns and prognoses for clear cell adenocarcinoma of the cervix (CCAC), a poorly understood rare tumor.

**Methods:**

A retrospective case–control study was conducted using the Surveillance, Epidemiology, and End Results (SEER) database, focusing on females diagnosed with CCAC between 2000 and 2019. Kaplan–Meier analysis, propensity score matching, Cox regression analysis, and subgroup analysis were used to assess treatment outcomes and risk factors.

**Results:**

Of the 52 153 patients with cervical cancer in the SEER database, 528 had CCAC. Overall survival (OS) was worse for patients with early-stage and locally advanced CCAC disease, although no differences in survival were observed for patients with stage IVB disease compared to those with other histologies. In our investigation into treatment patterns, we have discovered that surgical treatment was the preferred choice for the majority of patients with locally advanced CCAC (58.5%). Further, Kaplan–Meier analysis revealed that surgery improved OS in CCAC patients (65.6 vs. 25.3%, *P*=0.000), with similar results in locally advanced-stage patients (57.9 vs. 26.7%, *P*=0.000). Moreover, multivariate Cox regression analysis revealed that surgery was significantly associated with a more favorable prognosis in CCAC patients with locally advanced disease (HR 0.299, 95% CI: 0.153–0.585, *P*=0.000). Consistent findings were observed following propensity score matching (HR 0.283, 95% CI: 0.106–0.751, *P*=0.011). According to the subgroup analyses, surgical intervention continued to show a beneficial effect on CCAC patients with locally advanced disease (HR=0.31, 95% CI: 0.21–0.46, *P*<0.001). In particular, we also found that compared to patients who received primary radiotherapy (RT), those who underwent radical surgery exhibited a significantly prolonged OS in locally advanced CCAC patients. Furthermore, multivariate Cox regression analysis revealed that surgery was associated with better outcomes in patients with stage IB3-IIA2 and locally resectable stage IIIC patients (HR 0.207, 95% CI=0.043–0.991, *P*=0.049). However, this trend was not observed for patients with stage IIB-IVA (except locally resectable stage IIIC) CCAC.

**Conclusions:**

Surgery should be considered the preferred treatment option for patients with locally advanced CCAC at stage IB3-IIA2 and locally resectable stage IIIC.

## Background

Highlights

- This study aimed to analyze the relevant prognostic factors and the treatment patterns in patients with CCAC.
- Our findings validated a significant survival benefit from surgical resection in CCAC patients with locally advanced disease, particularly for the IB3-IIA2 and locally resectable stage IIIC subgroups.
- Surgery should be considered the preferred treatment option for patients with locally advanced CCAC at stage IB3-IIA2 and locally resectable stage IIIC.

Cervical cancer is one of the most common gynecological malignant tumors, ranking fourth in incidence and third in mortality worldwide in females^1^. Clear cell adenocarcinoma of the cervix (CCAC) is a rare type of cervical cancer reportedly associated with a history of exposure to diethylstilbestrol (DES)^2-3^, a synthetic estrogen derivative given to millions of pregnant women during the 1940s to 1970s. Since DES was banned in the 1970s, CCAC has become extremely rare, accounting for only 4–9% of cervical adenocarcinomas^4^. Currently, there is limited literature on the study of CCAC, primarily consisting of retrospective studies with insufficient data volume^5-10^, and conducting prospective randomized controlled studies poses significant challenges. Consequently, there are no specific treatment guidelines for CCAC; treatments are mainly analogous to those for common cancer types (squamous cell carcinoma (SCC), usual type adenocarcinoma (ACC), and adenosquamous carcinoma (ASC)). National Comprehensive Cancer Network (NCCN) guidelines^11^ for the common type of cervical cancer suggest that surgery is mainly used for early-stage cancer and that nonsurgical methods are mainly used as first-choice treatment for locally advanced cancer (LACC) (stage IB3-IVA). However, CCAC exhibits unique characteristics, such as insensitivity to radiation and chemotherapy^6,9-10^, and there are molecular biological distinctions from common type of cervical cancer, whether the current guidelines for common cancer types are suitable for CCAC lacks evidence-based support. Therefore, dedicated research is required to establish appropriate treatment strategies for CCAC. Additionally, Due to the paucity of available data from previous studies, our understanding regarding the prognosis of CCAC remains incomplete. Thus, retrospective studies on the treatment and prognosis of this rare cervical cancer type are warranted.

This retrospective analysis utilized the Surveillance, Epidemiology, and End Results (SEER) database to analyze relevant prognostic factors and treatment patterns in patients with CCAC.

## Methods

The SEER database was utilized to identify patients diagnosed with CCAC using SEER*Stat 8.4.2 software. The database ‘SEER Research Plus Data, 17 Registries, Nov 2021 sub (2000–2019)’ was searched for ‘Site and Morphology, Behavior recode for analysis= Malignant’, and ‘Site and Morphology, Site recode ICD-O-3/WHO 2008=Cervix Uteri’. Our search identified 55 738 women diagnosed with the following cervical cancer histology types based on International Classification of Diseases (ICD)-10 morphology codes: CCAC (8310), squamous cell carcinoma (SCC: 8070, 8071, 8072, 8073, 8076), adenocarcinoma (ACC: 8140), and adenosquamous carcinoma (ASC: 8560). We excluded women without histologic confirmation, for whom tumor stage information was missing or incomplete, whose survival duration was unknown, or whose surgery status was unknown. Thus, 52 153 cervical cancer patients were included in the SEER analysis. The clinicopathological variables for each patient in this analysis included age at diagnosis, year of diagnosis, race, FIGO 2018 stage, NCCN stage, tumor size, lymph node metastasis, surgery, radiation, type of radiation, chemotherapy and combination radiation, and chemotherapy. The original staging information of the included patients was assessed according to the SEER-modified AJCC stage 3rd, Derived SEER Combined Stage Group, Derived AJCC Stage Group, 6th and 7th, and Derived EOD 2018 Stage Group. Subsequently, we restaged the original staging information according to FIGO 2018 staging rules. After the re-staging, locally advanced cancer (LACC) is defined as stage IB3-IVA, while early stage is defined as stage IA1-IB2, and metastatic stage is defined as stage IVB. As the clinical information and data used in this SEER study were downloaded from a public database, ethical approval and informed consent were waived. This population-based cohort study was strictly conducted according to the strengthening the reporting of cohort, cross-sectional, and case–control studies in Surgery (STROCSS) criteria^12^ (Supplemental Digital Content 1, http://links.lww.com/JS9/D234).

### Statistical analysis

Statistical analysis was performed using IBM Corp SPSS v.21.0 software and R (version 4.3.2). In the SEER study population, categorical variables were expressed as counts and percentages. Comparisons between groups were analyzed using the *χ* ^2^ test. Hazard ratios (HRs) and 95% CIs for associations between histologic subtype and overall survival (OS) were estimated using Cox proportional hazards regression models, both in the overall study population and in subgroups stratified by stage (early, locally advanced, and metastatic). We also explored the associations between treatment and OS stratified by histologic subtype and adjusted for age and race. To reduce selection bias of baseline differences in clinical characteristics of the CCAC patients, we used 1:1 propensity score matching (PSM) method to match the patients in the surgery and nonsurgery groups, with a ratio of 1 and a caliper value of 0.2. The effect of treatment on OS was compared using the Kaplan–Meier method, and the results were compared by log-rank tests. Both univariate and multivariate Cox analyses were performed to control for confounders. All variables (age at diagnosis, year of diagnosis, race, tumor size, lymph node metastasis, surgery, radiation, and chemotherapy) were included in univariate Cox regression analyses, and variables that were significant or clinically considered to affect patient prognosis were included in multivariable Cox regression analyses. Seven predefined subgroup analyses were performed on baseline characteristics: race, age, type of radiation, chemotherapy, regional node positivity or negativity, NCCN stage and tumor size. Predefined subgroup analyses were performed for surgery (yes and no). Hazard ratios (HRs) are reported with 95% CIs. All tests were two-sided, and *P*<0.05 was considered to indicate statistical significance.

## Results

### Comparison of the clinical characteristics of patients with CCAC and other cervical cancer histologies.

Among 52 153 patients with cervical cancer diagnosed between 2000 and 2019, only 528 had CCAC. CCAC patients had a significantly older age of disease onset than did those with other pathological types (*P*=0.000), with a median age of 58.5 years, and the most affected age in CCAC group was over 65 years old (36.0%). In contrast, the majority of patients in the other three groups were aged 35 to 44 years (25.2% vs. 29.8% vs. 29.6%). Analysis based on race revealed a significant proportion of white patients across all histologic types (CCAC: 77.8%; SCC: 74%; ACC: 80.9%; and ASC: 77.2%, *P*=0.000). The stage distribution for CCAC was similar to that for other histologic types: 47.3% of patients had stage I disease at presentation, 13.4% had stage II disease, 22.0% had stage III disease, and 17.2% had stage IV disease. Similar results were obtained when considering NCCN staging. However, lymph node metastasis rates were higher in CCAC than in SCC and ACC but slightly lower than in ASC (CCAC: 14.6%; SCC: 8.5%; ACC: 6.8%; ASC: 17.2%, *P*=0.000) (Table 1).

**Table 1:** Characteristics of 52 153 women with cervical cancer overall and by histology.

	Overall	Clear cell adenocarcinoma	Squamous cell carcinoma	Adenocarcinoma	Adenosquamous carcinoma	*p*
	n=52 153	n=528	n=40 492	n=8924	n=2209	
Age at diagnosis						
≤24	512 (1.0)	34 (6.4)	411 (1.0)	47 (0.5)	20 (0.9)	**0.000**
25–34	7377 (14.1)	32 (6.1)	5702 (14.1)	1312 (14.7)	333 (15.0)	
35–44	13 586 (26.1)	66 (12.5)	10 204 (25.2)	2662 (29.8)	654 (29.6)	
45–54	12 336 (23.7)	87 (16.5)	9546 (23.6)	2150 (24.1)	553 (25.0)	
55–64	8928 (17.1)	119 (22.5)	7074 (17.5)	1387 (15.5)	348 (15.8)	
≥65	9414 (18.1)	190 (36.0)	7555 (18.7)	1366 (15.3)	303 (13.7)	
Year of diagnosis						
2000–2004	13 883 (26.6)	131 (24.8)	11 091 (27.4)	1991 (22.3)	670 (30.3)	**0.000**
2005–2009	12 840 (24.6)	125 (23.7)	9990 (24.7)	2102 (23.6)	623 (28.2)	
2010–2014	12 460 (23.9)	134 (25.4)	9580 (23.7)	2251 (25.2)	495 (22.4)	
2015–2019	12 970 (24.9)	138 (26.1)	9831 (24.3)	2580 (28.9)	421 (19.1)	
Race						
White	39 301 (75.4)	411 (77.8)	29 965 (74.0)	7220 (80.9)	1705 (77.2)	**0.000**
Black	6993 (13.4)	60 (11.4)	6048 (14.9)	652 (7.3)	233 (10.5)	
Other	5455 (10.5)	57 (10.8)	4164 (10.3)	967 (10.8)	267 (12.1)	
Unknown	404 (0.8)	0 (0.0)	315 (0.8)	85 (1.0)	4 (0.2)	
Stage						
I	25 726 (49.3)	250 (47.3)	18 647 (46.1)	5787 (64.8)	1042 (47.2)	**0.000**
II	7752 (14.9)	71 (13.4)	6464 (16.0)	908 (10.2)	309 (14.0)	
III	11 579 (22.2)	116 (22.0)	9783 (24.2)	1168 (13.1)	512 (23.2)	
IV	7096 (13.6)	91 (17.2)	5598 (13.8)	1061 (11.9)	346 (15.7)	
NCCN stage						
Confined to the cervix	24 982 (47.9)	231 (43.8)	18 139 (44.8)	5625 (63.0)	987 (44.7)	**0.000**
Locally advanced	20 123 (38.6)	207 (39.2)	16 798 (41.5)	2238 (25.1)	880 (39.8)	
Metastatic	7010 (13.4)	90 (17.0)	5523 (13.6)	1055 (11.8)	342 (15.5)	
Unknown	38 (0.1)	0 (0.0)	32 (0.1)	6 (0.1)	0 (0.0)	
Tumor size, cm						
≤4	14 550 (27.9)	161 (30.5)	10 659 (26.3)	2935 (32.9)	795 (36.0)	**0.000**
＞4	11 010 (21.1)	139 (26.3)	9118 (22.5)	1230 (13.8)	523 (23.7)	
Unknown	26 593 (51.0)	228 (43.2)	20 715 (51.2)	4795 (53.3)	891 (40.3)	
Lymph node metastasis						
No	14 764 (28.3)	229 (43.4)	9691 (23.9)	3979 (44.6)	865 (39.2)	**0.000**
Yes	4528 (8.7)	77 (14.6)	3462 (8.5)	609 (6.8)	380 (17.2)	
Unknown	32 861 (63.0)	222 (42.0)	27 339 (67.5)	4336 (48.6)	964 (43.6)	
Surgery						
None	22 337 (42.8)	174 (33.0)	18 905 (46.7)	2553 (28.6)	705 (31.9)	**0.000**
Local tumor excision	6491 (12.4)	48 (9.1)	5239 (12.9)	1014 (11.4)	190 (8.6)	
Extrafascial hysterectomy +/− BSO	12 600 (24.2)	156 (29.5)	9076 (22.4)	2788 (31.2)	580 (26.3)	
Radical hysterectomy	10 239 (19.6)	143 (27.1)	6885 (17.0)	2510 (28.1)	701 (31.7)	
Pelvic exenteration	193 (0.4)	4 (0.8)	145 (0.4)	31 (0.3)	13 (0.6)	
Unknown	293 (0.6)	3 (0.6)	242 (0.6)	28 (0.3)	20 (0.9)	
Regional lymph node surgery						
No	36 714 (70.4)	279 (52.8)	30 098 (74.3)	5106 (57.2)	1231 (55.7)	**0.000**
Yes	15 236 (29.2)	248 (47.0)	10 229 (25.3)	3787 (42.4)	972 (44.0)	
Unknown	203 (0.4)	1 (0.2)	165 (0.4)	31 (0.3)	6 (0.3)	
Local treatment						
Radical surgery						**0.000**
With RT	3451 (6.6)	69 (13.1)	2454 (6.1)	597 (6.7)	331 (15.0)	
Without RT/unknown	6788 (13.0)	74 (14.0)	4431 (10.9)	1913 (21.4)	370 (16.7)	
Primary RT						
EBRT	9552 (18.3)	83 (15.7)	8118 (20.0)	992 (11.1)	359 (16.3)	
EBRT + brachytherapy	10 836 (20.8)	76 (14.4)	9245 (22.8)	1175 (13.2)	340 (15.4)	
Brachytherapy	1626 (3.1)	13 (2.5)	1406 (3.5)	165 (1.8)	42 (1.9)	
Radiation with unknown details	267 (0.5)	1 (0.2)	238 (0.6)	19 (0.2)	9 (0.4)	
Other	19 633 (37.6)	212 (40.2)	14 600 (36.1)	4063 (45.5)	758 (34.3)	
Radiation						
No or unknown	23 036 (44.2)	204 (38.6)	16 584 (41.0)	5381 (60.3)	867 (39.2)	**0.000**
Yes	29 117 (55.8)	324 (61.4)	23 908 (59.0)	3543 (39.7)	1342 (60.8)	
Type of radiation						
No or unknown	23 036 (44.2)	204 (38.6)	16 584 (41.0)	5381 (60.3)	867 (39.2)	**0.000**
EBRT only	13 817 (26.5)	164 (31.1)	11 205 (27.7)	1683 (18.9)	765 (34.6)	
EBRT+brachytherapy	12 094 (24.7)	122 (23.1)	10 710 (26.4)	1578 (17.7)	494 (22.4)	
Other	2396 (4.6)	37 (7.2)	1993 (4.9)	282 (3.2)	83 (3.8)	
Chemotherapy						
No or Unknown	27 520 (52.8)	268 (50.8)	20 322 (50.2)	5870 (65.8)	1060 (48.0)	**0.000**
Yes	24 633 (47.2)	260 (49.2)	20 170 (49.8)	3054 (34.2)	1149 (52.0)	
Combination radiation and chemotherapy						
None or unknown	21 385 (41.0)	172 (32.6)	15 424 (38.1)	5008 (56.1)	781 (35.4)	**0.000**
Radiation only	6135 (11.8)	96 (18.2)	4898 (12.1)	862 (9.7)	279 (12.6)	
Chemotherapy only	1651 (3.2)	32 (6.1)	1160 (2.9)	373 (4.2)	86 (3.9)	
Radiation and chemotherapy	22 982 (44.1)	228 (43.2)	19 010 (46.9)	2681 (30.0)	1063 (48.1)	

*P*<0.05 was considered significant.

*p* values are representative of trends across groups.

BSO, bilateralsalpingo-oophorectomy; EBRT, external beam radiotherapy; NCCN, National Comprehensive Cancer Network; RT, radiotherapy.

### The treatment patterns and prognoses of CCAC differ from those of other cervical cancer histologies.

Early-stage CCAC patients had poorer OS than SCC, ACC, and ASC patients (SCC HR 0.543, 95% CI: 0.425–0.693; ACC HR 0.366, 95% CI: 0.284–0.471; ASC HR 0.603, 95% CI: 0.456–0.796, *P*=0.000), and this trend extended to patients with locally advanced disease. Patients with CCAC had worse OS than patients with SCC, ACC, or ASC (SCC HR 0.819, 95% CI: 0.681–0.985, *P*=0.034; ACC HR 0.840, 95% CI: 0.692–1.019, *P*=0.077; ASC HR 0.778, 95% CI: 0.633–0.957, *P*=0.018), although the differences from those with ACC were not statistically significant. Additionally, no differences in overall survival for patients with metastatic disease were observed between these histologic types (SCC HR 0.975, 95% CI: 0.770–1.235, *P*=0.835; ACC HR 1.073, 95% CI: 0.841–1.370, *P*=0.570; ASC HR 0.955, 95% CI: 0.735–1.242, *P*=0.732) (Supplemental Table 1, Supplemental Digital Content 2, http://links.lww.com/JS9/D235).

The majority of patients with early-stage CCAC opted for surgical intervention, similar to patients with other pathological types (CCAC 89.2%; SCC 85.4%; ACC 91%; ASC 89.8%). The proportion of CCAC patients who received adjuvant treatment was greater (46.3%) than that of patients with other histologies in the early stage (SCC 28.2%; ACC 20.8%; ASC 36.1%). For patients with locally advanced disease, surgical treatment was the preferred choice for the majority of patients with CCAC and ASC (CCAC 58.5%, ASC 58.4%). However, for SCC and ACC, nonsurgical treatment remains the preferred choice for most patients (SCC 68.9%, ACC 55.7%). Additionally, regardless of the pathological type, radiation combined with chemotherapy was the most common adjuvant therapy for patients with locally advanced disease (CCAC 66.7%; SCC 76.0%; ACC 70.2%; and ASC 74.9%). This finding was consistent with the treatment of patients with advanced metastasis (CCAC 48.9%; SCC 57.9%; ACC 42.7%; and ASC 56.1%) (Table 2).

**Table 2:** Treatment characteristics of 52 153 women with cervical cancer by stage stratified by histology.

	Treatment, *n* (%)*									
	Surgical treatment						Adjuvant treatment				
Histology	No surgery	Local tumor excision or destruction	Simple hysterectomy	Radical hysterectomy	Pelvic exenteration	Unknown	None or unknown	Radiation only	Chemotherapy only	Chemotherapy and radiation
Clear cell adenocarcinoma (528)	174 (33.0)	48 (9.1)	156 (29.5)	143 (27.1)	4 (0.8)	3 (0.6)	172 (32.6)	96 (18.2)	32 (6.1)	228 (43.2)
Early stage (*n*=231)	24 (10.4)	28 (12.1)	94 (40.7)	84 (36.4)	0 (0.0)	1 (0.4)	124 (53.7)	49 (21.2)	12 (5.2)	46 (19.9)
Locally advanced (*n*=207)	86 (41.5)	16 (7.7)	51 (24.6)	48 (23.2)	4 (1.9)	2 (1.0)	34 (16.4)	33 (15.9)	2 (1.0)	138 (66.7)
Metastatic (*n*=90)	64 (71.1)	4 (4.4)	11 (12.2)	11 (12.2)	0 (0.0)	0 (0.0)	14 (15.6)	14 (15.6)	18 (20.0)	44 (48.9)
Squamous cell carcinoma (40 492)	18 905 (46.7)	5239 (12.9)	9076 (22.4)	6885 (17.0)	145 (0.4)	242 (0.6)	15 424 (38.1)	4898 (12.1)	1160 (2.9)	19 010 (46.9)
Early stage (*n*=18 139)	2599 (14.3)	3498 (19.3)	7115 (39.2)	4866 (26.8)	21 (0.1)	40 (0.2)	13 029 (71.8)	1920 (10.6)	160 (0.9)	3030 (16.7)
Locally advanced (*n*=16 798)	11 577 (68.9)	1480 (8.8)	1670 (9.9)	1866 (11.1)	55 (0.3)	150 (0.9)	1493 (8.9)	2214 (13.2)	328 (2.0)	12 763 (76.0)
Metastatic (*n*=5523)	4709 (85.3)	256 (4.6)	286 (5.2)	151 (2.7)	69 (1.2)	52 (0.9)	892 (16.2)	762 (13.8)	671 (12.1)	3198 (57.9)
Unknown (*n*=32)	20 (62.5)	5 (15.6)	5 (15.6)	2 (6.3)	0 (0.0)	0 (0.0)	10 (31.3)	2 (6.3)	1 (3.1)	19 (59.4)
Adenocarcinoma (8924)	2553 (28.6)	1014 (11.4)	2788 (31.2)	2510 (28.1)	31 (0.3)	28 (0.3)	5008 (56.1)	862 (9.7)	373 (4.2)	2681 (30.0)
Early stage (*n*=5625)	504 (9.0)	796 (14.2)	2276 (40.5)	2037 (36.2)	4 (0.1)	8 (0.1)	4457 (79.2)	467 (8.3)	43 (0.8)	658 (11.7)
Locally advanced (*n*=2238)	1246 (55.7)	163 (7.3)	385 (17.2)	413 (18.5)	18 (0.8)	13 (0.6)	312 (13.9)	276 (12.3)	78 (3.5)	1572 (70.2)
Metastatic (*n*=1055)	802 (76.0)	54 (5.1)	123 (11.7)	60 (5.7)	9 (0.9)	7 (0.7)	236 (22.4)	118 (11.2)	251 (23.8)	450 (42.7)
Unknown (*n*=6)	1 (16.7)	1 (16.7)	4 (66.7)	0 (0.0)	0 (0.0)	0 (0.0)	3 (50.0)	1 (16.7)	1 (16.7)	1 (16.7)
Adenosquamous (2209)	705 (31.9)	190 (8.6)	580 (26.3)	701 (31.7)	13 (0.6)	20 (0.9)	781 (35.4)	279 (12.6)	86 (3.9)	1063 (48.1)
Early stage (*n*=987)	99 (10.0)	109 (11.0)	344 (34.9)	431 (43.7)	2 (0.2)	2 (0.2)	631 (63.9)	129 (13.1)	15 (1.5)	212 (21.5)
Locally advanced (*n*=880)	367 (41.7)	67 (7.6)	193 (21.9)	238 (27.0)	7 (0.8)	8 (0.9)	95 (10.8)	110 (12.5)	16 (1.8)	659 (74.9)
Metastatic (*n*=342)	239 (69.9)	14 (4.1)	43 (12.6)	32 (9.4)	4 (1.2)	10 (2.9)	55 (16.1)	40 (11.7)	55 (16.1)	192 (56.1)

* Row percentages.l.

### Baseline characteristics of eligible CCAC patients in the surgery and nonsurgery groups before and after PSM, as well as in the radical surgery and primary RT groups.

Compared to the nonsurgery group, the surgery group had smaller tumors (≤4 cm, 40.8 vs. 8.8%), more lymph node metastases (18.4 vs. 6.5%), in earlier stages (confined to the cervix, 58.1 vs. 13.5%) and a smaller proportion of patients receiving radiation therapy (54.2 vs. 76.5%) (Supplemental Table 2, Supplemental Digital Content 2, http://links.lww.com/JS9/D235). The same factors used for univariate analysis, as presented in supplemental Table 5 (Supplemental Digital Content 2, http://links.lww.com/JS9/D235) were employed for PSM analysis. The propensity scores for both groups exhibited significant overlap, with all covariates yielding *P* values greater than 0.05 for all stages of CCAC groups (Supplemental Table 5, Supplemental Digital Content 2, http://links.lww.com/JS9/D235) and for the locally advanced stage subgroup (Supplemental Table 3, Supplemental Digital Content 2, http://links.lww.com/JS9/D235). The baseline characteristics of eligible CCAC patients in the radical surgery and primary RT groups were presented in Supplemental Table 4 (Supplemental Digital Content 2, http://links.lww.com/JS9/D235).

### The impact of surgery on the prognosis of patients with CCAC.

**Figure 1:**
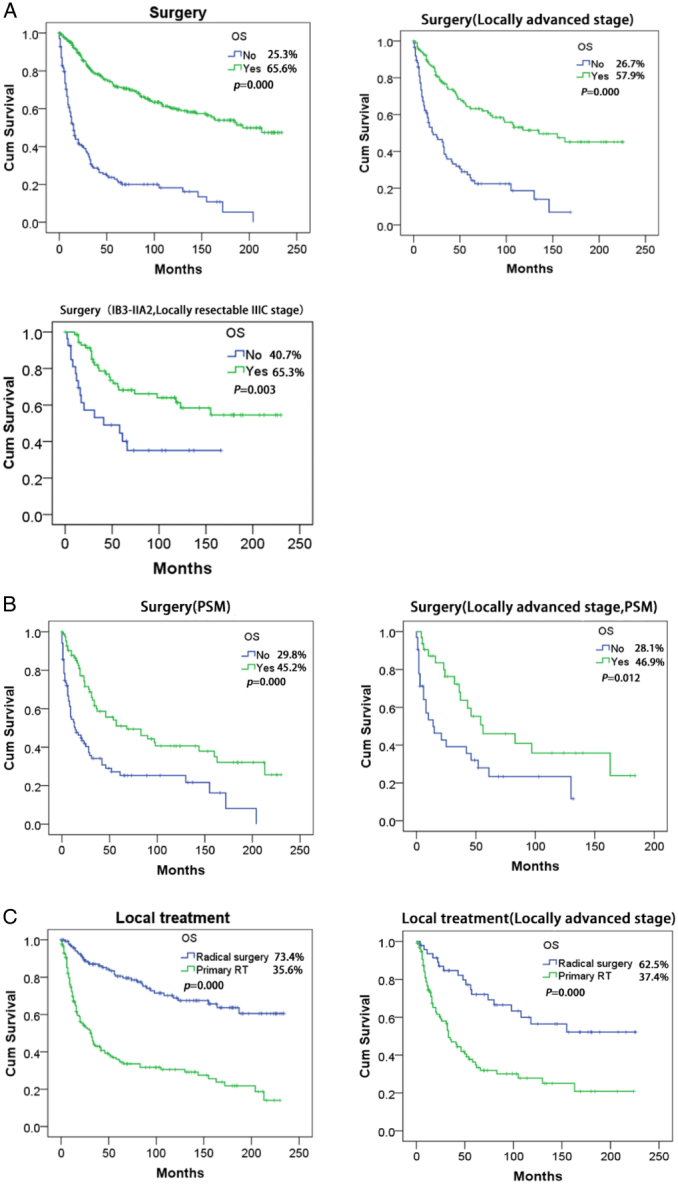
Overall survival analysis of CCAC patients in different subgroups. (A) OS analysis of all-stage, locally advanced, and stage IB3-IIA2, locally resectable stage IIIC CCAC patients in the surgery and nonsurgery groups. (B) OS analysis of all-stage and locally advanced-stage CCAC patients in the surgery and nonsurgery groups after PSM. (C) OS analysis of all-stage and locally advanced CCAC patients in the radical surgery and primary RT groups. *P*<0.05 was considered significant. OS, overall survival; PSM, propensity score matching; RT, radiotherapy.

The median follow-up time for surviving CCAC patients was 114 months (95% CI: 100–127 months) in the surgery group and 73 months (95% CI: 44–101 months) in the nonsurgery group. The OS outcomes for CCAC patients who underwent surgery were superior to those of patients who did not undergo surgery (65.6 vs. 25.3%, *P*=0.000). This difference was consistently observed among locally advanced patients (57.9 vs. 26.7%, *P*=0.000) and IB3-IIA2, locally resectable IIIC stage patients (65.3 vs. 40.7%, *P*=0.003) (Locally resectable IIIC stage: Cervical lesions were confined to the cervix without parametrial metastasis, but lymph node metastasis was present). According to the matched data after PSM, the OS of the surgery group was significantly greater than that of the nonsurgery group in all stages (45.2 vs. 29.8%, *P*=0.000), with similar results observed in those with a locally advanced stage (46.9 vs. 28.1%, *P*=0.012). Compared to those who underwent primary radiotherapy (RT), CCAC patients who underwent radical surgery had longer OS (73.4 vs. 35.6%, *P*=0.000), which was also observed in those with a locally advanced stage (62.5 vs. 37.4%, *P*=0.000) (Fig. 1).

### Prognostic factors influencing OS in patients with CCAC.

**Figure 2:**
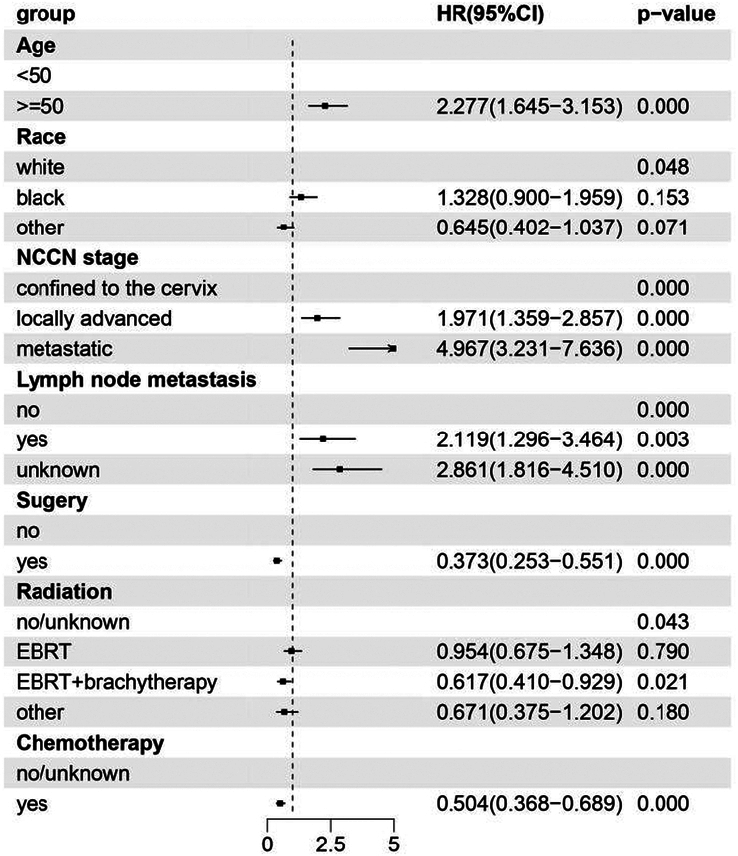
Forest plot for multivariate Cox regression analyses of factors associated with overall survival in all-stage CCAC patients. EBRT, external beam radiotherapy; HR, hazard ratio; NCCN, National Comprehensive Cancer Network.

We conducted univariate and multivariate Cox analyses to explore prognostic factors in patients with CCAC. According to univariate analysis, age at diagnosis, race, NCCN grade, tumor size, lymph node metastasis, surgery, radiation type, and chemotherapy were significantly associated with OS (*P*<0.05) (Supplemental Table 5, Supplemental Digital Content 2, http://links.lww.com/JS9/D235). Multivariate analysis revealed that patients with older age (HR 2.277, 95% CI: 1.645–3.153, *P*=0.000), higher NCCN stage (locally advanced HR 1.971, 95% CI: 1.359–2.857; metastatic HR 4.967, 95% CI: 3.231–7.636, *P*=0.000), and lymph node metastasis (HR 2.119, 95% CI: 1.296–3.464, *P*=0.003) had worse OS. Conversely, surgery (HR 0.373, 95% CI: 0.253–0.551, *P*=0.000), chemotherapy (HR 0.504, 95% CI: 0.368–0.689, *P*=0.000), and external beam (EBRT) combined with brachytherapy (HR 0.617, 95% CI: 0.410–0.929, *P*=0.021) were associated with improved OS (Fig. 2).

**Figure 3:**
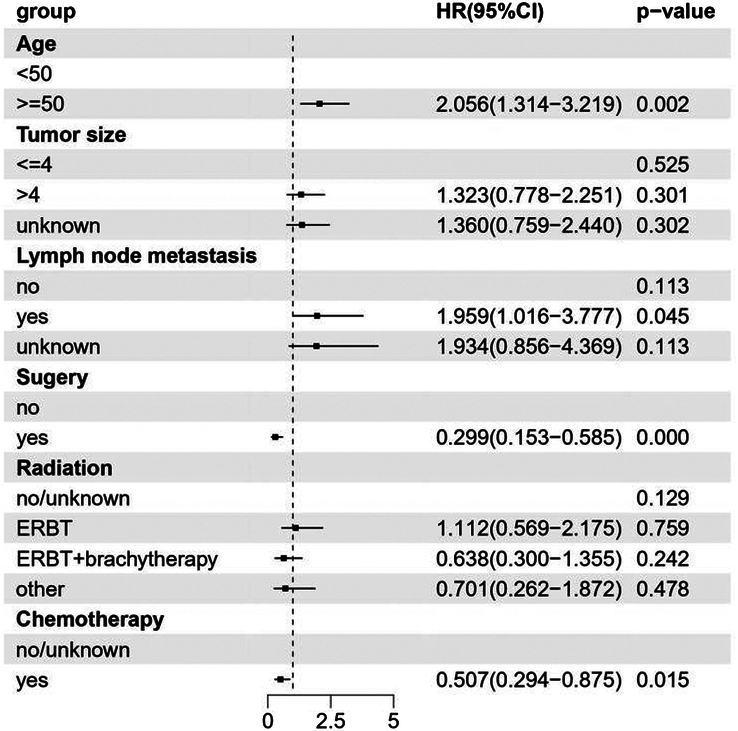
Forest plot for multivariate Cox regression analyses of factors associated with overall survival in locally advanced CCAC patients. EBRT, external beam radiotherapy; HR, hazard ratio.

For patients with locally advanced CCAC, univariate analysis revealed that age at diagnosis, tumor size, lymph node metastasis, and surgery were significantly associated with OS (*P*<0.05) (Supplemental Table 6, Supplemental Digital Content 2, http://links.lww.com/JS9/D235). Multivariate analysis revealed that patients derived significant benefits from surgery (HR 0.299, 95% CI=0.153–0.585, *P*=0.000) and chemotherapy (HR 0.507, 95% CI: 0.294–0.875, *P*=0.015). Conversely, lymph node metastasis (HR 1.959, 95% CI: 1.016–3.777, *P*=0.045) and older age (HR 2.056, 95% CI: 1.314–3.219, *P*=0.002) were risk factors for OS (Fig. 3). Multivariate analysis of propensity score-matched groups showed that patients could derive significant benefits from surgery in all stages (HR 0.397, 95% CI: 0.256–0.615, *P*=0.000) (Supplemental Table 7, Supplemental Digital Content 2, http://links.lww.com/JS9/D235), and in locally advanced stages as well (HR 0.283, 95% CI: 0.106–0.751, *P*=0.011) (Supplemental Table 8, Supplemental Digital Content 2, http://links.lww.com/JS9/D235).

Because locally advanced disease covers stages IB3 to IVA, we further divided the patients into IB3-IIA2, locally resectable stage IIIC subgroups and IIB-IVA (except locally resectable stage IIIC) subgroups. For patients with IB3-IIA2, locally resectable stage IIIC, multivariate analysis revealed that patients derived significant benefits from surgery (HR 0.207, 95% CI: 0.043–0.991, *P*=0.049) (Supplemental Table 9, Supplemental Digital Content 2, http://links.lww.com/JS9/D235). However, for patients with IIB-IVA (except locally resectable stage IIIC), the surgical intervention did not yield any discernible improvement in the overall outcome (HR 0.562, 95% CI: 0.275–1.148, *P*=0.114) (Supplemental Table 10, Supplemental Digital Content 2, http://links.lww.com/JS9/D235).

**Figure 4:**
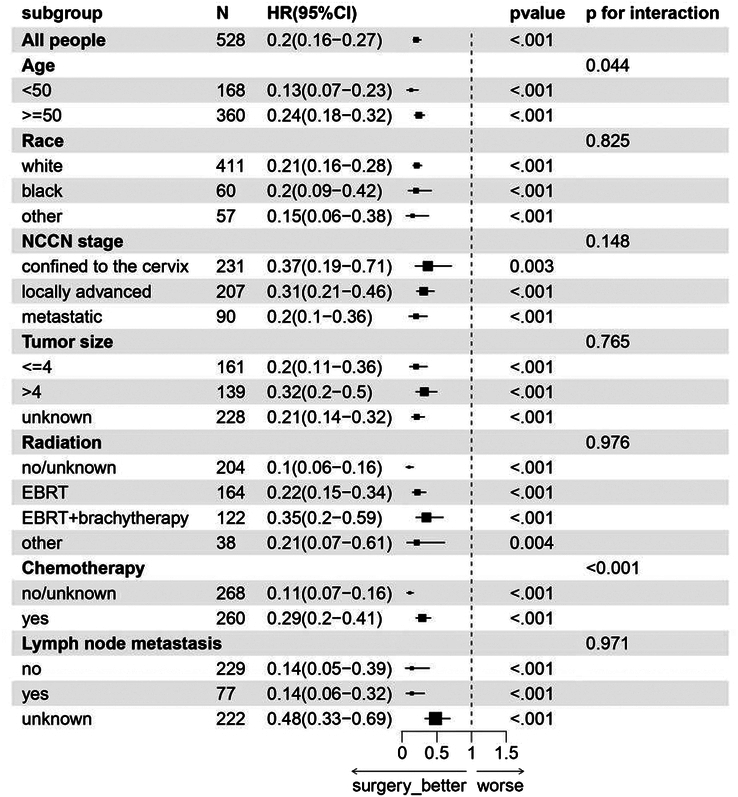
Forest plot of the subgroup analysis concerning overall survival. EBRT, external beam radiotherapy; HR, hazard ratio; N, number; NCCN National Comprehensive Cancer Network.

Furthermore, prespecified subgroup analyses were conducted to assess the effects of surgery on CCAC patients in different subgroups. We found that surgery was associated with a reduced risk of mortality in all subgroups (*P*<0.05), including the locally advanced subgroup (HR=0.31, 95% CI: 0.21–0.46, *P*<0.001). However, age and chemotherapy were significantly associated with surgery (*P* for interaction <0.05) (Fig. 4).

## Discussion

Due to the rarity of CCAC, prognostic factors and treatment strategies are inherently unpredictable, and there are no standard guidelines. The SEER database offers researchers a substantial sample size to identify factors associated with patient survival, enhancing the statistical power of studies on rare tumors. In this study, we evaluated the treatment patterns and prognosis of patients with CCAC based on the SEER database, and our findings further validated a significant survival benefit from surgical resection in CCAC patients with locally advanced disease, particularly for the IB3-IIA2 and locally resectable stage IIIC subgroups.

Our results revealed that patients with early-stage and locally advanced CCAC disease had worse OS than those with other histological types of cervical cancer. This finding is consistent with previous literature reports^8-9,13^, highlighting a relatively unfavorable prognosis associated with CCAC. Reich *et al*.^13^ reported that the 5-year survival rate of early-stage CCAC patients was 67%, which was slightly worse than that of nonclear cell adenocarcinoma (77%) and cervical squamous cell carcinoma (80%) patients. However, the sample size for the CCAC patients in this study was only 15 individuals. Thomas *et al*.^9^ studied 34 CCAC patients and reported that the 3-year survival rate for early-stage CCAC patients (stage I or IIA) was 91%, while it dropped to 22% for advanced-stage patients (stage III–IV). Given the limitations of small sample sizes in previous studies, our research findings represent the highest level of evidence currently available.

To explore the appropriate treatment for CCAC, we first compared the therapy pattern with other pathological types of cervical cancer, and interestingly we found that surgery was preferred for locally advanced CCAC patients, whereas nonsurgical treatment was more common among SCC and ACC patients. Furthermore, OS analyses revealed that surgical intervention led to a more favorable prognosis, particularly in patients with locally advanced CCAC. Notably, we also found that compared to patients who received primary RT, those with CCAC who underwent radical surgery exhibited a significantly prolonged OS in locally advanced CCAC patients. Patients in the IB3-IIA2 stage subgroup and IIB-IVA stage subgroup may receive varied treatments for common histological types of cervical cancer in accordance with NCCN guidelines^11^. Additionally, the treatment approach for locally resectable IIIC stage cervical cancer has been controversial. Prior to the publication of the 2018 FIGO staging system^14^ for cervical cancer, NCCN guidelines^15^ recommended radical hysterectomy for patients with locally resectable IIIC stage disease. After the publication of the 2018 FIGO staging system, some researchers^16-17^ still advocated surgery for locally resectable IIIC stage patients, while others^18-19^ suggested that radiotherapy and chemotherapy should be considered as first-line options. Therefore, we further stratified patients with locally advanced disease into IB3-IIA2, locally resectable stage IIIC subgroups and IIB-IVA stage (except locally resectable stage IIIC) subgroups. Based on multivariate Cox analysis, surgery demonstrated a protective effect in CCAC patients with stage IB3-IIA2 and locally resectable stage IIIC. However, this trend was not observed for patients with stage IIB-IVA (except locally resectable stage IIIC) CCAC. The loss of surgical protection in patients with stage IIB-IVA (except locally resectable stage IIIC) cancer may be associated with the occurrence of metastasis. And a larger retrospective study is needed to determine whether surgery affects the prognosis of this group of patients.

The NCCN guidelines^11^ recommend concurrent chemoradiotherapy as the first preferred option for individuals with locally advanced disease, which contradicts our findings. For locally advanced cervical cancer of common type, there have been high-level evidence^20^ to support that surgery and radiotherapy are equivalent as primary treatment. However, given that a majority of patients with locally advanced cervical cancer meet the radiotherapy criteria, postoperative radiotherapy becomes highly probable, leading to an increased risk of complications associated with dual treatment involving surgery and radiotherapy. Consequently, both NCCN guidelines^11^ and FIGO guidelines^21^ have long recommended chemoradiotherapy as the first-choice approach for managing locally advanced cervical cancer of common type. However, the guidelines do not provide a clear recommendation for clear cell carcinoma. Our study, for the first time, demonstrated that surgery can significantly improve the prognosis of patients with locally advanced CCAC, particularly those at stage IB3-IIA2 and locally resectable stage IIIC. We propose that the superiority of surgery may be attributed to the relative insensitivity of CCAC to radiotherapy and chemotherapy compared to SCC^6,9-10^. Additionally, surgical intervention reduces the tumor burden, thereby enhancing the efficacy of subsequent radiotherapy and chemotherapy. Furthermore, lymph node dissection during surgery improves clinical staging accuracy and facilitates appropriate guidance for adjuvant therapy, and this approach may have effectively excised larger lymph nodes that cannot be completely eliminated by radiotherapy, consequently influencing patient prognosis.

No study has reported the effects of surgery on CCAC patients, and we are the first to conduct a comprehensive analysis of the treatment methods for CCAC, and focus on treatment of surgery for locally advanced CCAC. CCAC belongs to a special type of ACC, which is associated with worse outcomes and is less responsive to radiotherapy or chemotherapy than SCC^22-23^. In fact, a subset of researchers advocated prioritizing surgical intervention as the primary therapeutic approach for locally advanced ACC. A SEER study^24^ demonstrated that surgery plus postoperative radiotherapy is associated with improved OS and Cancer-Specific Survival (CSS) in patients with locally advanced ACC when compared to primary radiotherapy. Some investigations^25^ have also indicated that surgery represents the most efficacious local treatment modality for individuals with advanced clinical stages of ACC, as well as for those with small cell cervical cancer^26^, which are insensitive to chemotherapy and radiotherapy, consistent with our results. Our study emphasizes the important role of surgery in locally advanced disease, particularly those at stage IB3-IIA2 and locally resectable stage IIIC. It offers novel insights into clinical practice and potentially influence therapeutic approaches for CCAC in a clinically significant manner. Given the rarity of CCAC, conducting prospective clinical research poses challenges; hence, a large-scale retrospective study holds substantial value in providing clinical treatment references. The findings of our study have the potential to guide the clinical treatment of CCAC.

We revealed that surgery, chemotherapy and external beam (EBRT) combined with brachytherapy were associated with improved OS, while locally advanced patients of CCAC only benefited from surgery and chemotherapy. Due to the lack of clear information from the SEER database about the specific dose and course of treatment for patients receiving radiotherapy or chemotherapy, further retrospective or prospective analyses are warranted to investigate the potential impact of radiotherapy and chemotherapy on the prognosis of patients with CCAC. For early-stage patients, some studies have found no necessity for using radiotherapy or chemotherapy following surgery in high-risk CCAC patients. Liu *et al*.^10^ retrospectively analyzed 42 patients with CCAC in a single center and reported that adjuvant radiotherapy or chemotherapy did not affect Progression Free Survival (PFS) or OS in early-stage patients with intermediate-risk factors. Thomas *et al*.^9^ reported that adjuvant radiotherapy did not affect PFS or OS in stage I or IIA patients with CCAC and negative lymph nodes, but suggested that advanced-stage CCAC should be treated with a combination of radiotherapy and chemotherapy.

In addition, we found that patients with older age, higher NCCN stage and lymph node metastasis had worse OS. In our study, patients with CCAC who were younger than 50 years exhibited superior OS. Previous study^27^ have demonstrated a correlation between older age and poor prognosis in cervical adenocarcinoma patients. Our findings align with these results. In Liu *et al*.’s study^10^, lymph node metastasis led to worse OS (*P*<0.05), while tumor size (>4 cm) did not exhibit any association with prognosis. Conversely, Hanselaar *et al*.’s research^27^ reported that tumor size (>4 cm) is an important negative prognostic factor for CCAC patients. In our study, lymph node metastasis was suggested to contribute to a poorer prognosis; however, we did not observe any impact of tumor size on OS. Notably, the study conducted by Hanselaar was published in the early years, with a limited number of cases included, and the patients were specifically CCAC patients exposed to DES. Therefore, it is imperative to validate the accuracy of these findings.

Several limitations of our research should be noted, some of which are inherent to all SEER database analyses. First, as this study is retrospective, it is difficult to avoid potential selection bias when we are appraising the effect of surgical interventions on CCAC patients or deriving causal inferences, and it need to be verified in prospective, multicenter, large-sample trials. Second, our study covers the period from 2000 to 2019, and some of the data may be outdated or missing. Moreover, there may be heterogeneity in the research object, methods, data sources, and other aspects. Although we tried to control for potential confounding factors through methods such as multivariate Cox analyses, subgroup analyses, and propensity score matching analyses, we cannot completely eliminate the possible effect of statistical bias on the results. Third, we could not determine the exact time difference between surgery and diagnosis, as these details were unavailable in the SEER database, which might have led to immortal time bias. Finally, the available data for cervical cancer patients in the SEER database lack crucial pathological information, such as edge status, lymphatic vascular space infiltration, and stromal infiltration. In addition, several important prognostic factors, such as relapse-free survival, whether applied immunotherapy, antiangiogenic agents and targeted therapy drugs, were not considered in this study, and these factors may impact the prognosis of patients with CCAC. Nonetheless, the substantial patient population represents a significant strength of this study. Indeed, our study is the largest retrospective cohort specifically investigating treatment patterns in CCAC patients to date. Moreover, our research first demonstrated that patients with locally advanced CCAC of stage IB3-IIA2 and locally resectable stage IIIC treated with surgery exhibit better OS outcomes, indicating that surgery is recommended as the preferred for these patients group. In the future, a comprehensive investigation into the prognostic impact of adjuvant radiotherapy and chemotherapy on patients undergoing surgery at different stages can be undertaken to provide detailed insights. Additionally, a multicenter retrospective study can further explore the comparative efficacy of radiotherapy and chemotherapy versus radical surgery as the primary treatment for locally advanced CCAC. We suggest that future studies carefully consider potential selection bias and immortal-time bias when including cases and try to control for their effects as much as possible.

## Conclusions

In conclusion, surgery might improve outcomes in patients with locally advanced CCAC at the IB3-IIA2 stage and those with locally resectable stage IIIC cancer. Therefore, surgery is recommended as the preferred treatment option for this patient group.

## Ethical approval

The clinical information and data used in this SEER study were all downloaded from a public database. Therefore, ethical approval were waived.

## Consent

The clinical information and data used in this SEER study were all downloaded from a public database. Therefore, informed consent were waived.

## Source of funding

This study was supported by Guangdong Basic and Applied Basic Research Foundation (2022A1515012432), Beijing Xisike Clinical Oncology Research Foundation (Y-Young2022--0145), Beijing Kanghua Foundation for the Development of Traditional Chinese and Western Medicine (KH-2021-LLZX-049), Sun Yat-sen Clinical Research Cultivating Program (SYS-C-202001), National Natural Science Foundation of China (81602290)and (81972433).

## Author contribution

H.L.: concept and design; J.L. and H.Q.: drafting of the manuscript; Z.L. and Y.Y.: critical revision of the manuscript for important intellectual content; J.L., H.Q., and Y.Y.: statistical analysis; L.W. and D.X.: administrative, technical, or material support; H.L.: supervision. All authors contributed in acquisition, analysis, or interpretation of data.

## Conflicts of interest disclosure

The authors no relevant financial disclosures and declare no conflicts of interest.

## Research registration unique identifying number (UIN)

1. Name of the registry: Research Registry.
2. Unique identifying number or registration ID: research-registry10057.
3. Hyperlink to your specific registration (must be publicly accessible and will be checked): https://www.researchregistry.com/browse-theregistry#home/registrationdetails/65e875a32d5b3c00280e9250/.

## Guarantor

Huaiwu Lu had full access to all of the data in the study and take responsibility for the integrity of the data and the accuracy of the data analysis.

## Data availability statement

The data that support the findings of this study are openly available in the SEER database.

## Provenance and peer review

Not commissioned, externally peer-reviewed.

## Supplementary Material
