# Supplementary material for: Treatment patterns and prognosis of patients with clear cell adenocarcinoma of the cervix: a population-based cohort study
Source: Int J Surg. 2024 Aug 2;111(1):20–30. doi: 10.1097/JS9.0000000000001997 (PMC11745625; doi:10.1097/JS9.0000000000001997)
Supplement: Supplementary file 2 [file js9-111-0020-s002.docx]

Supplemental Table 1. Multivariable-adjusted hazard ratios (HRs) and 95% confidence intervals for the association between histology and overall survival among women with cervical cancer by stage

| **Histology** | Deaths N(%)^⁎^ | HR(95% CI)^1^ | P | HR(95% CI)^2^ | P | HR(95% CI)^3^ | P |
| --- | --- | --- | --- | --- | --- | --- | --- |
| **Early stage** | 16.7(4166/24982) |  |  |  |  |  |  |
| Clear Cell Adenocarcinoma | 66(1.6) |  | **0.000** |  | **0.000** |  | **0.000** |
| Squamous cell carcinoma | 3251(78.0) | 0.543(0.425-0.693) | **0.000** | 0.761(0.596-0.972) | **0.029** | 0.739(0.579-0.943) | **0.015** |
| Adenocarcinoma | 649(15.6) | 0.366(0.284-0.471) | **0.000** | 0.549(0.426-0.708) | **0.000** | 0.558(0.433-0.720) | **0.000** |
| Adenosquamous | 200(4.8) | 0.603(0.456-0.796) | **0.000** | 0.881(0.667-1.164) | 0.374 | 0.878(0.665-1.160) | 0.361 |
| **Locally advanced** | 46.7(9399/20123) |  |  |  |  |  |  |
| Clear Cell Adenocarcinoma | 114(1.2) |  | 0.101 |  | 0.273 |  | 0.107 |
| Squamous cell carcinoma | 7836(83.4) | 0.819(0.681-0.985) | **0.034** | 0.855(0.710-1.028) | 0.096 | 0.852(0.708-1.025) | 0.089 |
| Adenocarcinoma | 1030(11.0) | 0.840(0.692-1.019) | 0.077 | 0.887(0.731-1.077) | 0.226 | 0.906(0.747-1.100) | 0.319 |
| Adenosquamous | 419(4.5) | 0.778(0.633-0.957) | **0.018** | 0.858(0.697-1.055) | 0.147 | 0.863(0.701-1.061) | 0.162 |
| **Metastatic** | 78.9(5531/7010) |  |  |  |  |  |  |
| Clear Cell Adenocarcinoma | 70(1.3) |  | 0.079 |  | 0.165 |  | 0.121 |
| Squamous cell carcinoma | 4348(78.6) | 0.975(0.770-1.235) | 0.835 | 1.013(0.800-1.283) | 0.917 | 0.992(0.783-1.256) | 0.946 |
| Adenocarcinoma | 839(15.2) | 1.073(0.841-1.370) | 0.570 | 1.102(0.8631.406) | 0.437 | 1.086(0.851-1.387) | 0.505 |
| Adenosquamous | 274(5.0) | 0.955(0.735-1.242) | 0.732 | 1.007(0.774-1.309) | 0.961 | 1.000(0.769-1.301) | 0.998 |

1.Unadjusted hazard ratios (HRs) and 95% confidence intervals (CIs).

2.Hazard ratios (HRs) and 95% confidence intervals (CIs) adjusted for age (<50, ≥50).

3.Hazard ratios (HRs) and 95% confidence intervals (CIs) adjusted for age (<50, ≥50) and race (White, Black, Other).

⁎Row percentages.

Supplemental Table 2. Baseline characteristics of eligible patients with clear cell carcinoma of the cervix, stratified by surgery status before and after propensity score matching.

|  | Total（n =528） | No surgery (n=170） | Surgery（n=358） | p | Total（n =168） | No surgery（n=84） | Surgery（n=84） | p |
| --- | --- | --- | --- | --- | --- | --- | --- | --- |
| Age at diagnosis |  |  |  |  |  |  |  |  |
| ＜50 | 168(31.8) | 47(27.6) | 121(33.8) | 0.156 | 50(29.8) | 22(26.2) | 28(33.3) | 0.311 |
| ≥50 | 360(68.2) | 121(33.8) | 237(66.2) |  | 118(70.2) | 62(73.8) | 56(66.7) |  |
| Year of diagnosis |  |  |  |  |  |  |  |  |
| 2000-2004 | 131(24.8) | 31(18.2) | 100(27.9) | 0.090 | 48(28.6) | 20(23.8) | 28(33.3) | 0.487 |
| 2005-2009 | 125(23.7) | 42(24.7) | 83(23.2) |  | 44(26.2) | 22(26.2) | 22(26.2) |  |
| 2010-2014 | 134(25.4) | 45(26.5) | 89(24.9) |  | 36(21.4) | 21(25.0) | 15(17.9) |  |
| 2015-2019 | 138(26.1) | 52(30.6) | 86(24.0) |  | 40(23.8) | 21(25.0) | 19(22.6) |  |
| Race |  |  |  |  |  |  |  |  |
| White | 411(77.8) | 138(81.2) | 273(76.3) | 0.365 | 129(76.8) | 64(76.2) | 65(77.4) | 0.970 |
| Black | 60(11.4) | 18(10.66) | 42(11.7) |  | 20(11.9) | 10(11.9) | 10(11.9) |  |
| Other | 57(10.8) | 14(8.2) | 43(12.0) |  | 19(11.3) | 10(11.9) | 9(10.7) |  |
| NCCN stage |  |  |  |  |  |  |  |  |
| Confined to the cervix | 231(43.8) | 23(13.5) | 208(58.1) | **0.000** | 51(30.4) | 23(27.4) | 28(33.3) | 0.237 |
| Locally advanced | 207(39.2) | 86(50.6) | 121(33.8) |  | 80(47.6) | 38(45.2) | 42(50.0) |  |
| Metastatic | 90(17.0) | 61(35.9) | 29(8.1) |  | 37(22.0) | 23(27.4) | 14(16.7) |  |
| Tumor size, cm |  |  |  |  |  |  |  |  |
| ≤4 | 161(30.5) | 15(8.8) | 146(40.8) | **0.000** | 29(17.3) | 9(10.7) | 20(23.8) | 0.056 |
| ＞4 | 139(26.3) | 66(38.8) | 73(20.4) |  | 64(38.1) | 37(44.0) | 27(32.1) |  |
| Unknown | 228(43.2) | 89(52.4) | 139(38.8) |  | 75(44.6) | 38(45.2) | 37(44.0) |  |
| Lymph node metastasis |  |  |  |  |  |  |  |  |
| No | 229(43.4) | 4(2.4) | 225(62.8) | **0.000** | 11(6.5) | 4(4.8) | 7(8.3) | 0.297 |
| Yes | 77(14.6) | 11(6.5) | 66(18.4) |  | 28(16.7) | 11(13.1) | 17(20.2) |  |
| Unknown | 222(42.0) | 155(91.2) | 67(18.7) |  | 129(76.8) | 69(82.1) | 60(71.4) |  |
| Radiation |  |  |  |  |  |  |  |  |
| No | 204(38.6) | 40(23.5) | 164(45.8) | **0.000** | 48(28.6) | 27(32.1) | 21(25.0) | 0.306 |
| Yes | 324(61.4) | 130(76.5) | 194(54.2) |  | 120(71.4) | 57(67.9) | 63(75.0) |  |
| Chemotherapy |  |  |  |  |  |  |  |  |
| No or unknown | 268(50.8) | 54(31.8) | 214(59.8) | **0.000** | 69(41.1) | 38(45.2) | 31(36.9) | 0.272 |
| Yes | 260(49.2) | 116(68.2) | 144(40.2) |  | 99(58.9) | 46(54.8) | 53(63.1) |  |

p < 0.05 was considered significant.

EBRT, External beam radiotherapy; NCCN:National Comprehensive Cancer Network

Supplemental Table 3. Baseline characteristics of eligible patients of locally advanced patients with clear cell carcinoma of the cervix, stratified by surgery status before and after propensity score matching.

|  | Total（n =207） | No surgery (n=86） | Surgery（n=121） | p | Total（n =64） | No surgery（n=32） | Surgery（n=32） | p |
| --- | --- | --- | --- | --- | --- | --- | --- | --- |
| Age at diagnosis |  |  |  |  |  |  |  |  |
| ＜50 | 69(33.3) | 26(30.2) | 43(35.5) | 0.425 | 16(25.0) | 6(18.8) | 10(31.3) | 0.248 |
| ≥50 | 138(66.7) | 60(69.8) | 78(64.5) |  | 48(75.0) | 26(81.3) | 22(68.8) |  |
| Year of diagnosis |  |  |  |  |  |  |  |  |
| 2000-2004 | 52(25.1) | 14(16.3) | 38(31.4) | **0.049** | 13(20.3) | 4(12.5) | 9(28.1) | 0.377 |
| 2005-2009 | 50(24.2) | 20(23.3) | 30(24.8) |  | 17(26.6) | 8(25.0) | 9(28.1) |  |
| 2010-2014 | 60(29.0) | 28(32.6) | 32(26.4) |  | 16(25.0) | 10(31.3) | 6(18.8) |  |
| 2015-2019 | 45(21.7) | 24(27.9) | 21(17.4) |  | 18(28.1) | 10(31.3) | 8(25.0) |  |
| Race |  |  |  |  |  |  |  |  |
| White | 166(80.2) | 75(87.2) | 91(75.2) | 0.102 | 46(71.9) | 25(78.1) | 21(65.6) | 0.126 |
| Black | 23(11.1) | 6(7.0) | 17(14.0) |  | 10(15.6) | 2(6.3) | 8(25.0) |  |
| Other | 18(8.7) | 5(5.8) | 13(10.7) |  | 8(12.5) | 5(15.6) | 3(9.4) |  |
| Tumor size, cm |  |  |  |  |  |  |  |  |
| ≤4 | **51(24.6)** | 10(11.6) | 41(33.9) | **0.001** | 14(21.9) | 7(21.9) | 7(21.9) | 0.960 |
| ＞4 | 84(40.6) | 42(48.8) | 42(34.7) |  | 29(45.3) | 15(46.9) | 14(43.8) |  |
| Unknown | 72(34.8) | 34(39.5) | 38(31.4) |  | 21(32.8) | 10(31.3) | 11(34.4) |  |
| Lymph node metastasis |  |  |  |  |  |  |  |  |
| No | 47(22.7) | 2(2.3) | 45(37.2) | **0.000** | 6(9.4) | 2(6.3) | 4(12.5) | 0.769 |
| Yes | 57(27.5) | 5(5.8) | 52(43.0) |  | 10(15.6) | 5(15.6) | 5(15.6) |  |
| Unknown | 103(49.8) | 79(91.9) | 24(19.8) |  | 48(75.0) | 25(78.1) | 23(71.9) |  |
| Radiation |  |  |  |  |  |  |  |  |
| No | 36(17.4) | 10(11.6) | 26(21.5) | 0.065 | 12(18.8) | 8(25.0) | 4(12.5) | 0.337 |
| Yes | 171(82.6) | 76(88.4) | 95(78.5) |  | 52(81.3) | 24(75.0) | 28(8.5) |  |
| Chemotherapy |  |  |  |  |  |  |  |  |
| No or unknown | 67(32.4) | 20(23.3) | 47(38.8) | **0.018** | 21(32.8) | 13(40.6) | 8(25.0) | 0.183 |
| Yes | 140(67.6) | 66(76.7) | 74(61.2) |  | 43(67.2) | 19(59.4) | 24(75.0) |  |

p < 0.05 was considered significant.

EBRT, External beam radiotherapy

Supplemental Table 4. Baseline characteristics of eligible patients with clear cell carcinoma of the cervix, stratified by local treatment status

|  | Total（n =528） | Radical surgery （n=143） | Primary RT（n=173） | p |
| --- | --- | --- | --- | --- |
| Age at diagnosis |  |  |  |  |
| ＜50 | 112(35.4) | 58(40.6) | 54(31.2) | 0.084 |
| ≥50 | 204(64.4) | 85(59.4) | 119(68.8) |  |
| Year of diagnosis |  |  |  |  |
| 2000-2004 | 81(25.6) | 45(31.5) | 36(20.8) | 0.188 |
| 2005-2009 | 76(24.1) | 31(21.7) | 45(26.0) |  |
| 2010-2014 | 69(21.8) | 330(21.0) | 39(22.5) |  |
| 2015-2019 | 90(28.5) | 37(25.9) | 53(30.6) |  |
| Race |  |  |  |  |
| White | 247(78.2) | 109(76.2) | 138(79.8) | 0.091 |
| Black | 35(11.1) | 13(9.1) | 22(12.7) |  |
| Other | 34(10.8) | 21(14.7) | 13(7.5) |  |
| NCCN stage |  |  |  |  |
| Confined to the cervix | 113(35.8) | 84(58.7) | 29(16.8) | **0.000** |
| Locally advanced | 147(46.5) | 48(33.6) | 99(57.2) |  |
| Metastatic | 56(17.7) | 11(7.7) | 45(26.0) |  |
| Tumor size, cm |  |  |  |  |
| ≤4 | 83(26.3) | 62(43.4) | 21(12.1) |  |
| ＞4 | 100(31.6) | 30(21.0) | 70(40.5) | **0.000** |
| Unknown | 133(42.1) | 51(35.7) | 82(47.4) |  |
| Lymph node metastasis |  |  |  |  |
| No | 116(36.7) | 103(72.0) | 13(7.5) | **0.000** |
| Yes | 51(16.1) | 36(25.2) | 15(8.7) |  |
| Unknown | 149(47.2) | 4(2.8) | 145(83.8) |  |
| Chemotherapy |  |  |  |  |
| No or unknown | 128(40.5) | 87(60.8) | 41(23.7) | **0.000** |
| Yes | 188(59.5) | 56(39.2) | 132(76.3) |  |

p < 0.05 was considered significant.

EBRT, External beam radiotherapy; RT:Radiotherapy; NCCN:National Comprehensive Cancer Network

Supplemental Table 5. Univariable Cox regression analysis for overall survival in patients with clear cell carcinoma of the cervix

|  | Univariable | | |
| --- | --- | --- | --- |
| Characteristics | HR | CI(95%） | p |
| Age at diagnosis |  |  |  |
| <50 |  |  |  |
| ≥50 | 2.560 | 1.871-3.502 | **0.000** |
| Year of diagnosis |  |  |  |
| 2000-2004 |  |  | 0.403 |
| 2005-2009 | 1.255 | 0.896-1.759 | 0.187 |
| 2010-2014 | 1.242 | 0.878-1.758 | 0.221 |
| 2015-2019 | 1.368 | 0.908-2.062 | 0.133 |
| Race |  |  |  |
| White |  |  | **0.021** |
| Black | 1.411 | 0.980-2.031 | 0.064 |
| other | 0.647 | 0.408-1.025 | 0.064 |
| NCCN stage |  |  |  |
| Confined to the cervix |  |  | **0.000** |
| Locally advanced | 2.518 | 1.857-3.414 | **0.000** |
| Metastatic | 7.537 | 5.312-10.692 | **0.000** |
| Tumor size, cm |  |  |  |
| ≤4 |  |  | **0.000** |
| ＞4 | 2.317 | 1.668-3.218 | **0.000** |
| Unknown | 2.202 | 1.600-3.031 | **0.000** |
| Lymph node metastasis |  |  |  |
| No |  |  | **0.000** |
| Yes | 3.006 | 1.999-4.520 | **0.000** |
| Unknown | 5.490 | 4.023-7.492 | **0.000** |
| Surgery |  |  |  |
| No | 0.207 | 0.160-0.268 | **0.000** |
| Yes |  |  |  |
| Radiation |  |  |  |
| No/ Unknown |  |  | **0.001** |
| EBRT | 1.807 | 1.338-2.440 | **0.000** |
| EBRT+brachytherapy | 1.325 | 0.947-1.854 | 0.100 |
| Other | 1.145 | 0.669-1.961 | 0.621 |
| Chemotherapy |  |  |  |
| No or Unknown |  |  |  |
| Yes | 1.367 | 1.064-1.756 | **0.014** |

p < 0.05 was considered significant.

EBRT, External beam radiotherapy; HR,Hazard ratio; CI, confidence interval

Supplemental Table 6. Univariable Cox regression analysis for overall survival in locally advanced patients with clear cell carcinoma of the cervix.

|  | Univariable | | |
| --- | --- | --- | --- |
| Characteristics | HR | CI(95%） | p |
| Age at diagnosis |  |  |  |
| ＜50 |  |  |  |
| ≥50 | 1.836 | 1.193-2.825 | **0.006** |
| Year of diagnosis |  |  |  |
| 2000-2004 |  |  | 0.584 |
| 2005-2009 | 1.326 | 0.806-2.179 | 0.267 |
| 2010-2014 | 1.271 | 0.773-2.090 | 0.344 |
| 2015-2019 | 1.473 | 0.786-2.760 | 0.227 |
| Race |  |  |  |
| White |  |  | 0.220 |
| Black | 1.261 | 0.742-2.148 | 0.391 |
| other | 0.573 | 0.266-1.237 | 0.156 |
| Tumor size, cm |  |  |  |
| ≤4 |  |  | **0.026** |
| ＞4 | 1.819 | 1.129-2.931 | **0.014** |
| Unknown | 1.892 | 1.129-3.169 | **0.016** |
| Lymph node metastasis |  |  |  |
| No |  |  | **0.000** |
| Yes | 1.697 | 0.920-3.133 | 0.091 |
| Unknown | 3.518 | 2.057-6.016 | **0.000** |
| Surgery |  |  |  |
| No |  |  |  |
| Yes | 0.316 | 0.216-0.462 | **0.000** |
| Radiation |  |  |  |
| No/ Unknown |  |  | 0.655 |
| EBRT | 0.992 | 0.593-1.660 | 0.977 |
| EBRT+brachytherapy | 0.859 | 0.502-1.472 | 0.581 |
| Other | 0.628 | 0.267-1.479 | 0.287 |
| Chemotherapy |  |  |  |
| No or Unknown |  |  |  |
| Yes | 0.782 | 0.532-1.147 | 0.208 |

p < 0.05 was considered significant.

EBRT, External beam radiotherapy; HR,Hazard ratio; CI, confidence interval

Supplemental Table7. Multivariable Cox regression analysis for overall survival in all stage patients with clear cell carcinoma of the cervix after propensity score matching.

|  | Multivariable | | |
| --- | --- | --- | --- |
| Characteristics | HR | CI(95%） | p |
| Age at diagnosis |  |  |  |
| ＜50 |  |  |  |
| ≥50 | 1.935 | 1.149-3.260 | **0.013** |
| Year of diagnosis |  |  |  |
| 2000-2004 |  |  | 0.735 |
| 2005-2009 | 1.224 | 0.729-2.057 | 0.445 |
| 2010-2014 | 1.250 | 0.673-2.318 | 0.480 |
| 2015-2019 | 0.894 | 0.448-1.788 | 0.752 |
| Race |  |  |  |
| White |  |  | 0.154 |
| Black | 1.502 | 0.817-2.759 | 0.190 |
| other | 0.640 | 0.308-1.331 | 0.232 |
| NCCN stage |  |  |  |
| Confined to the cervix |  |  | **0.000** |
| Locally advanced | 2.506 | 1.409-4.454 | **0.002** |
| Metastatic | 7.567 | 3.873-14.782 | **0.000** |
| Tumor size, cm |  |  |  |
| ≤4 |  |  | 0.330 |
| ＞4 | 0.945 | 0.506-1.765 | 0.859 |
| Unknown | 1.384 | 0.703-2.728 | 0.347 |
| Lymph node metastasis |  |  |  |
| No |  |  | 0.351 |
| Yes | 1.571 | 0.469-5.258 | 0.464 |
| Unknown | 2.022 | 0.686-5.962 | 0.202 |
| Surgery |  |  |  |
| No |  |  |  |
| Yes | 0.397 | 0.256-0.615 | **0.000** |
| Radiation |  |  |  |
| No/ Unknown |  |  | 0.232 |
| EBRT | 0.845 | 0.479-1.493 | 0.563 |
| EBRT+brachytherapy | 0.627 | 0.342-1.150 | 0.131 |
| Other | 0.337 | 0.093-1.216 | 0.097 |
| Chemotherapy |  |  |  |
| No or Unknown |  |  |  |
| Yes | 0.574 | 0.359-0.917 | **0.020** |

p < 0.05 was considered significant.

EBRT, External beam radiotherapy; NCCN:National Comprehensive Cancer Network; HR,Hazard ratio; CI, confidence interval

Supplemental Table 8. Multivariable Cox regression analysis for overall survival in locally advanced propensity score matching after propensity score matching .

|  | Multivariable | | |
| --- | --- | --- | --- |
| Characteristics | HR | CI(95%） | p |
| Age at diagnosis |  |  |  |
| ＜50 |  |  |  |
| ≥50 | 0.921 | 0.317-2.675 | 0.879 |
| Year of diagnosis |  |  |  |
| 2000-2004 |  |  | 0.574 |
| 2005-2009 | 1.627 | 0.510-5.189 | 0.411 |
| 2010-2014 | 0.735 | 0.239-2.260 | 0.591 |
| 2015-2019 | 1.076 | 0.298-3.891 | 0.911 |
| Race |  |  |  |
| White |  |  | 0.496 |
| Black | 1.618 | 0.500-5.239 | 0.422 |
| Other | 0.507 | 0.122-2.101 | 0.349 |
| Tumor size, cm |  |  |  |
| ≤4 |  |  | 0.207 |
| ＞4 | 0.510 | 0.200-1.303 | 0.159 |
| Unknown | 1.271 | 0.375-4.314 | 0.700 |
| Lymph node metastasis |  |  |  |
| No |  |  | 0.536 |
| Yes | 0.511 | 0.082-3.191 | 0.472 |
| Unknown | 0.409 | 0.082-2.040 | 0.276 |
| Surgery |  |  |  |
| No |  |  |  |
| Yes | 0.283 | 0.106-0.751 | **0.011** |
| Radiation |  |  |  |
| No/ Unknown |  |  | **0.002** |
| EBRT | 0.166 | 0.052-0.530 | **0.002** |
| EBRT+brachytherapy | 0.091 | 0.024-0.344 | **0.000** |
| Other | 0.030 | 0.003-0.311 | **0.003** |
| Chemotherapy |  |  |  |
| No or Unknown |  |  |  |
| Yes | 0.717 | 0.266-1.933 | 0.511 |

p < 0.05 was considered significant.

EBRT, External beam radiotherapy; HR,Hazard ratio; CI, confidence interval

Supplemental Table 9. Multivariable Cox regression analysis for overall survival in stage IB3-IIA2,Locally resectable IIIC patients.

|  | Multivariable | | |
| --- | --- | --- | --- |
| Characteristics | HR | CI(95%） | p |
| Age at diagnosis |  |  |  |
| ＜50 |  |  |  |
| ≥50 | 3.507 | 1.649-7.459 | **0.001** |
| Race |  |  |  |
| White |  |  | 0.498 |
| Black | 1.361 | 0.519-3.568 | 0.531 |
| other | 0.488 | 0.108-2.209 | 0.352 |
| Tumor size, cm |  |  |  |
| ≤4 |  |  | 0.935 |
| ＞4 | 0.867 | 0.400-1.880 | 0.718 |
| Unknown | 0.952 | 0.305-2.971 | 0.932 |
| Lymph node metastasis |  |  |  |
| No |  |  | 0.980 |
| Yes | 1.056 | 0.413-2.703 | 0.909 |
| Unknown | 0.889 | 0.162-4.871 | 0.893 |
| Surgery |  |  |  |
| No |  |  |  |
| Yes | 0.207 | 0.043-0.991 | **0.049** |
| Radiation |  |  |  |
| No/ Unknown |  |  | 0.358 |
| EBRT | 2.380 | 0.834-6.794 | 0.105 |
| EBRT+brachytherapy | 1.320 | 0.426-4.086 | 0.630 |
| Other | 1.714 | 0.377-7.783 | 0.485 |
| Chemotherapy |  |  |  |
| No or Unknown |  |  |  |
| Yes | 0.645 | 0.293-1.421 | **0.277** |

p < 0.05 was considered significant.

EBRT, External beam radiotherapy; HR, Hazard ratio; CI, confidence interval

Supplemental Table 10. Multivariable Cox regression analysis for overall survival in stage IIB-IVA (except locally resectable IIIC) patients.

|  | Multivariable | | |
| --- | --- | --- | --- |
| Characteristics | HR | CI(95%） | p |
| Age at diagnosis |  |  |  |
| ＜50 |  |  |  |
| ≥50 | 1.751 | 0.974-3.149 | 0.061 |
| Race |  |  |  |
| White |  |  | 0.480 |
| Black | 0.845 | 0.391-1.824 | 0.668 |
| other | 0.541 | 0.191-1.529 | 0.246 |
| Tumor size, cm |  |  |  |
| ≤4 |  |  | 0.582 |
| ＞4 | 1.468 | 0.672-3.206 | 0.336 |
| Unknown | 1.558 | 0.646-3.761 | 0.324 |
| Lymph node metastasis |  |  |  |
| No |  |  | 0.705 |
| Yes | 1.282 | 0.495-3.323 | 0.609 |
| Unknown | 1.473 | 0.594-3.651 | 0.403 |
| Surgery |  |  |  |
| No |  |  |  |
| Yes | 0.562 | 0.275-1.148 | 0.114 |
| Radiation |  |  |  |
| No/ Unknown |  |  | 0.160 |
| EBRT | 0.592 | 0.244-1.433 | 0.245 |
| EBRT+brachytherapy | 0.368 | 0.142-0.955 | **0.040** |
| Other | 0.399 | 0.098-1.620 | 0.199 |
| Chemotherapy |  |  |  |
| No or Unknown |  |  |  |
| Yes | 0.512 | 0.262-0.997 | **0.049** |

p < 0.05 was considered significant.

EBRT, External beam radiotherapy; HR, Hazard ratio; CI, confidence interval
